# The Genetics of Response to and Side Effects of Lithium Treatment in Bipolar Disorder: Future Research Perspectives

**DOI:** 10.3389/fphar.2021.638882

**Published:** 2021-03-25

**Authors:** Fanny Senner, Mojtaba Oraki Kohshour, Safa Abdalla, Sergi Papiol, Thomas G. Schulze

**Affiliations:** ^1^ Institute of Psychiatric Phenomics and Genomics (IPPG), University Hospital, LMU Munich, Munich, Germany; ^2^ Department of Psychiatry and Psychotherapy, University Hospital, LMU Munich, Munich, Germany; ^3^ Department of Immunology, Faculty of Medicine, Ahvaz Jundishapur University of Medical Sciences, Ahvaz, Iran; ^4^ Department of Pharmacology, Faculty of Pharmacy, University of Khartoum, Khartoum, Sudan; ^5^ Department of Psychiatry and Behavioral Sciences, SUNY Upstate Medical University, Syracuse, NY, United States

**Keywords:** lithium, bipolar disorder, pharmacogenetics, linkage and segregation studies, candidate-gene association studies, GWAS, treatment response, side effect

## Abstract

Although the mood stabilizer lithium is a first-line treatment in bipolar disorder, a substantial number of patients do not benefit from it and experience side effects. No clinical tool is available for predicting lithium response or the occurrence of side effects in everyday clinical practice. Multiple genetic research efforts have been performed in this field because lithium response and side effects are considered to be multifactorial endophenotypes. Available results from linkage and segregation, candidate-gene, and genome-wide association studies indicate a role of genetic factors in determining response and side effects. For example, candidate-gene studies often report GSK3β, brain-derived neurotrophic factor, and SLC6A4 as being involved in lithium response, and the latest genome-wide association study found a genome-wide significant association of treatment response with a locus on chromosome 21 coding for two long non-coding RNAs. Although research results are promising, they are limited mainly by a lack of replicability and, despite the collaboration of consortia, insufficient sample sizes. The need for larger sample sizes and “multi-omics” approaches is apparent, and such approaches are crucial for choosing the best treatment options for patients with bipolar disorder. In this article, we delineate the mechanisms of action of lithium and summarize the results of genetic research on lithium response and side effects.

## Introduction

Bipolar disorder (BD) is a common affective disorder with a high burden worldwide that leads to impaired quality of life and disability (Merikangas et al., 2011). The disease is characterized by mood changes from mania to depression and a 20-fold higher suicide risk than in the general population (Ösby et al., 2001). Clinical guidelines recommend mood stabilizers as the first-line treatment in BD. Lithium was first introduced in 1949. It is the oldest and still most frequently prescribed mood stabilizer in BD and has also proven effective in reducing suicidal ideation and suicide attempts (CADE, 1949; Rybakowski, 2014). Nevertheless, 30–55% of individuals treated with lithium do not benefit from it and experience side effects (Burgess et al., 2001). Furthermore, a recent meta-analysis revealed that about 50–60% of patients with bipolar mania do not respond sufficiently to lithium and require a change to another mood-stabilizing drug (Yildiz et al., 2011; Sienaert et al., 2013). After receiving lithium treatment for a first episode of psychotic mania, only 59% of patients are fully recovered after 12 months (Conus et al., 2004). As a result of insufficient response to lithium, polypharmacy, e.g., the combination of mood stabilizers and antipsychotics, has become more frequent and may even become standard care in BD (Baldessarini et al., 2008; Weinstock et al., 2014; Fung et al., 2019). A European survey revealed an increasing trend to treat bipolar depression with 3 (37.0%) or 4 (6.4%) drugs (Greil et al., 2012). However, polypharmacy and side effects lead to poorer adherence (Murray and Kroenke, 2001; Tareke et al., 2018; Semahegn et al., 2020), which in turn leads to poorer clinical outcomes (Fung et al., 2019). Many other mood stabilizers and antipsychotics are metabolized via the liver’s cytochrome P450 system, either as a substrate, inhibitor, or inducer. Therefore, polypharmacy with mood stabilizers and antipsychotics increases the risk that interactions will increase or decrease serum levels of these drugs. Lithium is an exception because its renal metabolism bypasses the cytochrome P450 system (Stahl, 2017); however, not to be neglected are its specific side effects, e.g., renal failure (Stahl, 2017). BD is a chronic disease, so most patients require long-term or even life-long mood-stabilizing treatment. Against this background, knowledge of the mode of action and prediction of response to and side effects of lithium and other drugs prescribed in BD is highly relevant. Research findings indicate a high heritability of and genetic involvement in lithium response, and studies have comprehensively investigated potential biomarkers (Mendlewicz et al., 1978; Grof et al., 2002; Kleindienst et al., 2005; Sussulini et al., 2009). A recent genome-wide association study (GWAS) provided insight into a single locus of four linked single-nucleotide polymorphisms (SNPs) on chromosome 21 that met genome-wide significance criteria for association with lithium response (Hou et al., 2016).

Despite the encouraging findings in this field, we are still far from identifying a clinical biomarker tool to predict lithium response and side effects. This lack of a biomarker can be explained by the lack of knowledge of the exact biological underpinnings of BD, the relevant molecular pathways of lithium, and the complex phenotypic and genetic architecture of BD and lithium response (Cruceanu et al., 2011; Hill et al., 2013). Psychiatric disorders are highly polygenic, and traits are extremely complex and heterogeneous. Moreover, response to pharmacotherapy is considered to be attributed to not only genomics but also the interaction of multiple genes and environmental factors.

In other medical fields, precision medicine is becoming more and more crucial and essential in clinical practice. Although psychiatry has expanded the focus on precision medicine, it needs to catch up with other specialties (Amare et al., 2017). So far, no tools are available for selecting the most appropriate drug for the individual patient with BD. Precision medicine should ensure that individuals receive the right treatment at the right time and should tailor treatment to the individual’s characteristics on the basis of genetic, environmental, and lifestyle factors (König et al., 2017). To identify the factors that we need to know about an individual patient, we have to classify individuals into subpopulations that differ in their susceptibility to a disease, biology, prognosis, and treatment response. The scientific basis of these strategies should represent a “pan-omics” approach that includes genomics, proteomics, metabolomics, and transcriptomics (Beckmann and Lew, 2016). To implement precision medicine in lithium treatment, we have to understand the action of lithium. An integrative approach seems to be necessary because lithium acts via multiple pathways. As a first step in translational medicine (i.e., from bench to bedside), we need to acquire a solid understanding of lithium’s pharmacological mechanisms in animal models. Then, we can transfer findings to clinical psychiatry and research. This article aims to provide a comprehensive overview of the developments in this field by delineating the pharmacological mechanisms of lithium observed in animal models and summarizing genetic research on lithium response and side effects. From this basis, we can derive future research topics in this field.

## Pharmacological Mechanisms of Action of Lithium in Animal Models

Lithium exerts its action through multiple signaling pathways and cellular processes (Jope, 1999). It is suggested to act by normalizing elevated levels of residual sodium during episodes of depression and mania (Coppen, 1967). Studies in the 1970s suggested a genetic influence on the distribution of lithium between intracellular and extracellular compartments (lithium ratio) (O’Connell et al., 1991). Later, many studies indicated that the lithium ratio does not affect treatment response but that it could determine the occurrence of side effects (Alda, 2015). Lithium was found to affect many neurotransmitters; however, most research has focused on catecholamines and acetylcholine (Jope, 1999). Unfortunately, these studies did not provide sufficient insight into the mechanisms of action of lithium. Thus, researchers started to study additional neurotransmitters, signal transduction pathways, and second messengers affected by lithium (Malhi et al., 2013). In animals, lithium was found to increase levels of serotonin by activating its synthesis and release and downregulating serotonin receptors (Wood et al., 1994). These pathways are assumed to play an important role in its anti-suicidal and antidepressant effects (Price and Heninger, 1994). The antimanic and antipsychotic effects of lithium are mediated by inhibiting the increased dopaminergic activity during manic phases (Alda, 2015). However, some results from animal models were conflicting: Some studies found no change in basal dopamine concentration after chronic lithium treatment, whereas others showed decreased dopamine concentrations during lithium treatment (Gambarana et al., 1999; Beaulieu et al., 2004; Ferrie et al., 2005; Can et al., 2016). Even so, sudden lithium withdrawal in rats was followed by increased dopamine levels, causing manic episodes (Ferrie et al., 2005). On another note, lithium was found to increase the synaptic concentration of glutamate and to stimulate N-methyl-D-aspartate (NMDA) (Dixon and Hokin, 1997); glutamate is an excitatory amino acid neurotransmitter that affects synaptic plasticity, learning, and memory. However, under experimental conditions lithium can act as an exotoxin, which leads to neurotoxicity (Nonaka et al., 1998). Stimulation of NMDA results in the accumulation of inositol 1, 4, 5-triphosphates (Dixon and Hokin, 1997). After long-term use, lithium exerts neuroprotective activity, probably by increasing glutamate reuptake and downregulating NMDA receptors and inositol 1, 4, 5- phosphates receptors (Nonaka et al., 1998), and in the short term it inhibits the inositol-mono phosphatase enzyme, resulting in inositol depletion and disruption of the inositol cycle (Ackermann et al., 1987; Malhi et al., 2013). Lithium competes with magnesium to inhibit G-protein–mediated hydrolysis of phosphoinositides (Jope, 1999); it affects G-protein by reducing G-protein receptor coupling and stabilizing G-protein’s inactive conformation (Manji and Lenox, 2000). The effect of lithium on glutamate might also be due to its effect on the inhibitory neurotransmitter gamma aminobutyric acid (GABA). Research in animals indicated that lithium decreases GABA turnover and increases GABA receptors (Ghasemi and Dehpour, 2011). Moreover, it exerts neuroprotective activity against apoptosis induced by many factors, including growth factor withdrawal, β-amyloid (Aβ), colchicine, high potassium deprivation, heat shock exposure, supra-therapeutic concentrations of anticonvulsants (phenytoin and carbamazepine), and glutamate-induced excitotoxicity (Chiu and Chuang, 2010). Furthermore, lithium was shown to act by modifying and inhibiting signaling of some second messengers (Alda, 2015). In stress, levels of transcription activator protein cAMP response element binding protein (CREB) and CREB-mediated transcription increase, and these increases are blocked by lithium (Böer et al., 2008). The neurotrophin brain-derived neurotrophic factor (BDNF) is vital for cortical development, synaptic plasticity, and neuronal survival and is assumed to be one of the mediators of the clinical effects of antidepressants (Chiu and Chuang, 2010). Chronic treatment with lithium leads to an increase in the expression of BDNF in rat brain (Hashimoto et al., 2004; Malhi et al., 2013). Moreover, research indicated that in rats pretreatment with lithium or BDNF protects cortical neurons against glutamate excitotoxicity (Hashimoto et al., 2004). In therapeutic concentrations, lithium was shown to inhibit the enzyme glycogen synthase kinase 3 (GSK3), which is responsible for gene expression, embryonic development, the circadian rhythm, glycogen synthesis, and neuronal survival (O’Brien and Klein, 2009). In a rodent model, inhibition of GSK3 was found to be involved in the antimanic and antidepressant activity of lithium (Chiu and Chuang, 2010).

## Pharmacogenetic Studies in Bipolar Disorder

### Lithium Response

Similar to many other psychiatric traits, clinical response to lithium treatment is considered to be a complex multifactorial phenotype. Family based studies suggested that long-term lithium treatment is a familial trait, showing that a remarkable proportion of patients who stabilize on lithium belong to lithium-responsive families (Grof et al., 2002; Duffy et al., 2007). This evidence led some authors to suggest that responders to lithium constitute a distinct heritable subtype of bipolar disorder characterized by a different disease course and clinical profile than patients responding to other mood stabilizers (Alda et al., 2005). Family based studies also showed that the offspring of lithium responders have a higher risk of developing BD than the offspring of non-responders (Duffy et al., 1998, Duffy et al., 2002). Taken together, these results indicate a clear role of genetic factors in determining lithium response. Since the early 1980s, molecular genetics has been trying to identify the specific genetic factors involved in this phenotype.

#### Linkage and Segregation Studies

To identify linkage and segregation studies on lithium treatment, we queried PubMed with the search string “linkage and lithium and response and bipolar.” A summary of the relevant studies identified in this search is shown in Table 1. All these studies were developed under the hypothesis that lithium-responsive patients represent a subgroup with heritable disease, as described above. However, none of the studies that explored a candidate genomic region found suggestive evidence of a linkage between genetic markers at these loci and lithium response (Turecki et al., 1998; Turecki et al., 1999a; Turecki et al., 1999b; Alda et al., 2000; Duffy et al., 2000). One study did find a significant linkage between (CA)n repeat in the locus encoding phospholipase C-gamma 1 (*PLCG1*); however, the observed maximum logarithm of the odds (LOD) score (1.45) was far from convincing (Turecki et al., 1998).

**TABLE 1:** Linkage and segregation studies in lithium response.

Author/Year	Sample size	Results	Remarks
Goldin et al. (1983)	18 families	No evidence for linkage	Segregation analysis in pedigrees with positive response to lithium using 21 autosomal markers
Turecki et al. (1998)	13 families	Modest linkage of (CA)_n_ repeat in *PLCG1* gene max LOD score = 1.45 under a dominant model	Linkage analysis of excellent lithium responders in families with unilineal transmission
Turecki et al. (1999a)	25 families	No evidence for linkage in the *MAOA* locus	Linkage analysis of excellent lithium responders
Turecki et al. (1999b)	19 families	No evidence for linkage in chromosome 18	Linkage analysis of excellent lithium responders using 11 dinucleotide markers covering chromosome 18
Alda et al. (2000)	24 families	No evidence for linkage in *CRH* and *PENK loci*	Linkage analysis of excellent lithium responders
Duffy et al. (2000)	24 families	No evidence for linkage in *GABRA3*, *GABRA5* and *GABRB3 loci*	Linkage analysis of excellent lithium responders
Radhakrishna et al. (2001)	1 large pedigree with 13 lithium-treated patients	Linkage with bipolar affective disorder in 20p11.2–q11.2 (max LOD score = 4.34 under 100% penetrance)	Linkage analysis based on 230 highly informative markers covering the entire genome. Full remission with lithium treatment in the affected members of the pedigree
Turecki et al. (2001)	31 families	Linkage on 15q14 locus, LOD score = 3.43. Suggestive results on 7q11.2 locus, LOD score = 2.68	Linkage analysis of excellent lithium responders. Linkage analysis based on a genome scan using 378 markers
Lopez de Lara et al. (2010)	36 families	Linkage on 14q11.2 locus, LOD score = 3.19 under the recessive model with lower penetrance. Suggestive results on 3p25.1 (LOD score = 2.53) and 3p14.1 (LOD score = 2.04)	Linkage analysis of excellent lithium responders. Linkage analysis based on a genome scan using 800 microsatellite markers
Umehara et al. (2020)	A Japanese family with lithium-responsive bipolar disorder consisting of 21 members	Linkage on 8p23.1 to 8p11.1; highest LOD score of 2.3 for marker rs10503492 on 8p22*. DOCK5* gene is located in the region with the highest LOD score	Genome-wide two-points linkage analysis using a 100K SNP array and microsatellite markers was performed on six affected family members, ten unaffected family members, and two family members with unknown status

LOD, logarithm of the odds.

Some of the studies performed genome scans of the entire genome (Goldin et al., 1983; Radhakrishna et al., 2001; Turecki et al., 2001; Lopez de Lara et al., 2010) and provided evidence of linkage (LOD score >3.0) with lithium-responsive bipolar disorder in 20p11.2–q11.2, 15q14, and 14q11.2 (Radhakrishna et al., 2001; Turecki et al., 2001; Lopez de Lara et al., 2010). In a new study in 2020, Umehara et al. performed a genome-wide linkage analysis on a Japanese family with lithium responder phenotype and reported that probably the region linked to BD is within ∼30 Mb section spanned by rs10503492 and rs10504053 on chromosomes 8p23.1–8p11.1, with the highest LOD score of 2.3 for rs10503492 on chromosome 8p22. They pointed to the *DOCK5* gene in this region and suggested that it may play a role in BD pathology (Umehara et al., 2020). However, each of these analyses identified different loci. This lack of replicability suggests that no major gene(s) is involved in lithium response, which is a familiar scenario in psychiatric phenotypes, where genetic analysis is hampered by the limited power of linkage studies to detect genetic factors of modest effect (Risch and Merikangas, 1996).

#### Candidate-Gene Association Studies

Genetic association studies, which are better powered to capture genetic effects, have also attempted to identify the genetic markers that modulate lithium response. Therefore, we queried PubMed with the search string “lithium and association and gene and bipolar” and classified the results into two groups: “case-control analyses of lithium-treated bipolar patients vs. healthy controls” and “case-only analyses of lithium-treated bipolar patients.” A summary of the candidate-gene association studies on lithium response published to date is shown in Supplementary Table S1. Many studies repeatedly investigated the effects of similar or different variants of same genes that are suspected to play a role in lithium response. To summarize these comprehensive results, we selected those genes for which at least two studies found the same results. Same results here mean the existence or absence of association for similar and different variants of the same gene with lithium response. Table 2 shows the characteristics of the studies that did not find evidence for an association of the gene(s) and lithium response in patients with BD, and Table 3 shows the characteristics of the studies that did. By comparing Tables 2, 3 one can see that the genes *GSK3β*, *BDNF*, and *SLC6A4* (serotonin transporter) seem to be involved bilaterally in poor and good response to lithium in different studies. Some of these studies showed no significant association with lithium response for the following gene variants: *BDNF* gene, G196A/Val66Met (Masui et al., 2006; Michelon et al., 2006; Wang et al., 2014) and rs10835210, rs11030101, rs11030102, rs12273363, rs2030324, rs2049045, rs6265”, rs7103411, rs962369, and rs988748 (Drago et al., 2010); *SLC6A4* gene, s/s, s/l, and l/l (Serretti et al., 2004; Masoliver et al., 2006; Michelon et al., 2006; Manchia et al., 2009); and *GSK3β* gene, rs3755557 (-1727A/T) (Michelon et al., 2006; Numajiri et al., 2012; Iwahashi et al., 2014), rs334558 (-50T/C) (Szczepankiewicz et al., 2006; Iwahashi et al., 2014), and rs11921360-A (Mitjans et al., 2015). On the other hand, other studies did find an association of the following variants of *GSK3β*, *BDNF*, and *SLC6A4* with lithium response in patients with BD: *GSK3β* gene, rs334558 (−50T/C) (Adli et al., 2007; Benedetti et al., 2012; Numajiri et al., 2012; Lin et al., 2013; Mitjans et al., 2015, 3), rs6438552 (McCarthy et al., 2011), rs3755557 (-1727A/T) (Iwahashi et al., 2014), rs1732170-rs1192136 (Mitjans et al., 2015); *BDNF* gene, Val66Met rs6265 (Rybakowski et al., 2005; Dmitrzak-Weglarz et al., 2008; Rybakowski et al., 2012; Wang et al., 2012; Wang et al., 2014) and rs988748 (Dmitrzak-Weglarz et al., 2008); and *SLC6A4* gene, l/l and s/s (Serretti et al., 2001; Benedetti et al., 2012), s allele and 10 repeat allele of STin2 (STin2.10) (Tharoor et al., 2013), and ins/del 44 pz (Rybakowski et al., 2012).

**TABLE 2:** Characteristics of gene studies that found no evidence for effect on lithium response. Only those genes are listed where at least two studies reported the same outcome.

Gene	Full name	Investigated variants	Number of studies	Total sample size	
				Cases	Controls
*GSK3β*	Glycogen synthase kinase–3β	rs11921360, rs334558, rs3755557	5	425	0
*BDNF*	Brain-derived neurotrophic factor	rs10835210, rs11030101, rs11030102, rs12273363, rs2030324, rs2049045, rs6265, rs7103411, rs962369, rs988748	4	658	288
*SLC6A4 or SERT, 5HTT, 5HTTLPR, HTTLPR*	Serotonin transporter	s/s, s/l, I/I	4	470	0
*HTR2A or 5HT2A*	Serotonin receptors 2 A	rs6311, rs6313	3	348	0
*DRD2*	Dopamine receptor D2	rs1799732, VNTR, Ser311Cys, NcoI, TaqIA	3	381	0
*DRD3*	Dopamine receptor D3	rs6280, 1/1, 1/2, 2/2, Mscl	3	299	0
*DRD4*	Dopamine receptor D4	rs1800955, 2 4, 4 4, 4 7	2	226	0
*CLOCK*	Circadian locomotor output cycle kaput	rs1801260, rs3736544, rs34897046, rs3805148, rs6849474, rs11932595, rs12648271, rs6850524, rs12649507, rs4340844, rs534654	2	397	0
*PER3*	Period circadian clock 3	rs2304672, rs228729, rs228642, rs228666, rs228697, rs2859388, rs2640909, rs836755, rs228727, rs10864315, rs4908694, rs228682, rs2172563, rs10462021	2	397	0
*MAOA*	Monoamine oxidase A	30-bp repeat	2	298	108
*MMP-9*	Matrix metallopeptidase 9	rs3918242	2	210	0
*NTRK2*	Neurotrophic receptor tyrosine kinase 2	rs1187326, rs2289656, rs1187327	2	209	0
*GNAL*	G protein subunit alpha L	A > G in intron 3 and T > G in intron 10	2	204	94
*IMPA2*	Inositol monophosphatase 2	−461C > T, -241_-237dup, −207 T > C, −185 A > G, 97–15G > A, 159 T > C, 230 + 141G > A, 382–44G > A, 443G > A, 490 + 13_14insA, rs3786282, 599 + 97G > A, 599 + 99G > A	2	164	0

**TABLE 3:** Characteristics of gene studies with evidence for effect on lithium response. Only those genes are listed where at least two studies reported the same outcome.

Gene	Full name	Investigated variants	Number of studies	Total sample size	
				Cases	Controls
*GSK3β*	Glycogen synthase kinase–3β	rs334558, rs6438552, rs3755557, rs1732170-rs1192136	7	770	131
*BDNF*	Brain-derived neurotrophic factor	rs6265, rs988748	5	919	674
*SLC6A4 or SERT, 5HTT, 5HTTLPR, HTTLPR*	Serotonin transporter	l/l, s/s, s allele, STin2.10, ins/del 44 pz	4	512	124
*CACNG2*	Calcium voltage-gated channel auxiliary subunit gamma 2	rs2284017, rs2284018, rs5750285, rs140040, rs2283967	2	712	0
*NR1D1*	Nuclear receptor subfamily 1 group D member 1	rs231433, rs2071427	2	452	0
*DRD1*	Dopamine receptor D1	rs4532	2	193	0

To summarize, an important number of research efforts were unable to provide pioneering insight into the association between candidate genes or variants and lithium response. The contradictory results demonstrate the need for additional research with larger sample sizes to provide powerful proof for factors that predict lithium response in patients with BD. Of relevance is that some of the studies described above led to the discovery of different genetic associations that can improve understanding of the genetic underpinnings of response to lithium and lead to the development of further hypotheses for additional studies. Nevertheless, similar to the situation in other research fields, replicability is one of the most important requirements to ensure that any approach accurately reflects nature's reality.

#### Genome-Wide Association Studies

As is the case with other complex diseases, our understanding of the genetic architecture of psychiatric disorders has improved dramatically over the last few decades thanks to advances in technologies, methods, and approaches (Gratten et al., 2014). GWASs try to identify common genetic variants with small effect sizes throughout the genome in large sample sizes that include cases and controls. To date, several GWASs have been performed on lithium response phenotypes in BD, and some of these studies have reported SNPs that showed significant associations with response. We identified relevant GWASs by querying PubMed with the search string “GWAS and lithium and bipolar.”

Song et al. reported that the SNP heritability (*h*2) of the lithium response phenotype for the subjective (self-reported) and objective (clinically documented) definitions of lithium response were 29 and 25%, respectively (Song et al., 2016). In a GWAS on 294 samples from people of Han Chinese descent in 2014, Chen et al. identified two SNPs (rs17026688 and rs17026651) in intronic regions of the *GADL1* (glutamate decarboxylase like protein 1) gene that showed a robust association with lithium response. They confirmed their results in a follow-up cohort of 100 patients and concluded that these SNPs have a sensitivity of 93% and can therefore be considered as predictors for the lithium response phenotype (Chen et al., 2014). Interestingly, in the same year the Consortium on Lithium Genetics (ConLiGen) performed a replication study on 218 samples with similar ancestry origin and not only did not confirm the findings reported by Chen et al., in 2014, but reported opposite results (Consortium on Lithium Genetics et al., 2014). A GWAS by Hou et al. on a sample of more than 2,500 patients identified four linked SNPs on a single locus on chromosome 21 (rs79663003, rs78015114, rs74795342, and rs75222709) that were associated with lithium response (Hou et al., 2016). Two of these SNPs, rs74795342 and rs75222709, are located in the intronic region of an identified long non-coding RNA (lncRNA) gene (AL157359.3) on chromosome 21, and the other two are located between this lncRNA gene and another lncRNA gene (AL157359.4) on the same chromosome. Decreased expression level of one of these lncRNAs (AL157359.3) appear to be found after acute episodes of BD (Hou et al., 2016).

In contrast to the GWASs described above, others failed to identify a significant association between candidate genetic variants and the lithium response phenotype, probably because they used different definitions of the lithium response phenotype and their sample sizes were not large enough to capture the suspected effect of SNPs. For example, in a GWAS on participants in the Systematic Treatment Enhancement Program for Bipolar Disorder (STEP-BD) cohort and the University College London cohort no SNPs reached the predefined threshold (Perlis et al., 2009); however, the unclear definition of the lithium response phenotype (intermediate and good responders were both considered to be “positive responders” and were compared with poor responders, who were classified as “negative responders”) and the small sample size (359 patients) can be considered as limitations of the study. In another study of samples recruited in Sweden and United Kingdom, a GWAS was first performed on self-reported lithium response patients (2,698 cases) and clinically documented ones (1,176 cases), and then a GWAS was conducted to compare lithium-responsive patients with healthy controls; this meta-analysis found no significant associations, but in the comparison of the responder patients with healthy controls an intronic SNP, rs116323614, in the gene *SESTD1* (SEC14 and spectrin domain one located on 2q31.2) met the GWAS threshold (Song et al., 2016).

One way to overcome the current problems in most GWASs is to form joint international research groups on a particular research area to facilitate large sample sizes and phenotype uniformity and to improve statistical and analytical power. A prominent example of this type of collaboration is the ConLiGen, which was founded in 2008 to create the largest sample size to date and conduct GWASs of lithium response in patients with BD (Schulze et al., 2010; ConLiGen, n.d.). Today, ConLiGen includes 39 countries on six continents and attempts to perform high quality, powerful GWASs on lithium response phenotype data (ConLiGen).

### Side Effects of Lithium

To examine whether studies have found associations between genetics and side effects of lithium, we queried PubMed with the search string “lithium and association and gene and bipolar and side effects.” The search identified 29 articles; however, after excluding non-human, non-bipolar, and review studies only three articles remained. These studies investigated the genes *GNAL* (G protein subunit alpha L), *GSK3β*, and *ACCN1* (acid sensing ion channel neurona-1) (Zill et al., 2003; Rybakowski et al., 2013; Tsermpini et al., 2017). However, they did not investigate all side effects known to occur with lithium treatment. Genetic underpinings of important side effects such as electrocardiogram changes, euthyroid goiter, hypothyroidism, hyperparathyroidism, extrapyramidal side effects, hyperreflexia, or myoclonus have not been assessed. The relevant data are summarized in Table 4. The limited search results indicate a clear lack of scientific studies on this topic. More research is needed to understand the mechanisms by which lithium’s action in treating BD leads to side effects. Additionally, further studies are needed to shed more light on the association of side effects with genetic variants to elucidate the severity and diversity of the harm caused by lithium treatment in patients.

**TABLE 4:** Genetic studies on side effects of lithium in patients with bipolar disorder.

Author/Year	Sample size	Gene	Results	Side effects analyzed
Zill et al. (2003)	149 patients with BD treated with lithium	*GNAL* (G protein subunit alpha L)	No evidence for association of 2 intronic SNPs (A > G in intron 3 and T > G in intron 10	Hand tremor, weight gain, cognitive dysfunction
Rybakowski et al. (2013)	78 patients with BD treated with lithium	*GSK3β* (glycogen synthase kinase-3β)	-50 C/T polymorphism; patients homozygous for C allele had significantly higher urine specific gravities	Alteration or impairment of: Urine specific gravity, serum creatinine, eGFR, serum neutrophil gelatinase-associated lipocalin, urine beta2-microglobulin
Tsermpini et al. (2017)	70 patients with BD treated with lithium	*ACCN1* (acid sensing ion channel Neurona-1)	rs378448; low eGFR associated with the CC genotype at this SNP	eGFR

BD, bipolar disorder; eGFR, estimated glomerular filtration rate; SNP, single-nucleotide polymorphism.

## Future Research Perspectives

Despite the recommendations to use lithium as a first-line treatment and the vast body of research in this field, knowledge is still lacking on the biological underpinnings of treatment response and side effects. The findings of genetic research are pioneering and represent a milestone in this field. However, the pharmacogenetic approaches applied to date are insufficient to obtain answers on the genetic architecture of lithium response and susceptibility to side effects. Pharmacogenetic research is unlikely to be able to develop predictive models (Middeldorp and Wray, 2018). Therefore, “multi-omic” approaches are required and could lead to a better understanding of lithium’s mechanisms of action. The collaboration of multiple working groups on this topic has made it possible to perform analyses on a large number of cases. Compared with samples in other areas, the sample sizes in genetic studies on BD are already enormous. Nevertheless, we must assume that the studies are still statistically underpowered. Also, replication studies are missing. Furthermore, one should note that when analyzing findings researchers must pay attention to patient ethnicity because it seems to be one of the important factors influencing treatment outcome (Lin et al., 1986). Various research efforts are aiming to solve these issues. For example, the well-established ConLiGen consortium is continuing to collect a significant number of samples. Likewise, other local and international initiatives are addressing the development of lithium-relevant biomarkers. BipoLife (www.bipolife.org) is a German project investigating genes and microRNAs with different lymphoblastoid cell line (LCL) expression profiles (Bipolife, n.d.). Larger, international consortia include the Pharmacogenomics of Bipolar Disorder (PGBD) study, which is collecting a 2-years prospective cohort of approximately 700 lithium-treated patients (Oedegaard et al., 2016), and the recently established European Response to Lithium Network (R-LiNK) (www.r-link.eu.com), which is pursuing the research question of lithium response in a multi-methodical manner by comprehensively characterizing a 2-years prospective cohort with deep phenotyping, “blood-omics,” magnetic resonance imaging, and Li7-magnetic resonance spectroscopy (Scott et al., 2019; R-LiNK, n.d.).

Besides answering the questions about treatment response and susceptibility to side effects, knowledge on other aspects is essential for understanding BD treatment in clinical practice. Research evidence, including a recent meta-review, indicates that lithium reduces the risk of suicidality (Lewitzka et al., 2015; Smith and Cipriani, 2017; Tondo and Baldessarini, 2018). The fact that this characteristic is unique to lithium underscores the importance of investigating the biological underpinnings and, consequently, the prediction of lithium’s anti-suicidal effects. To briefly summarize the overview published by Malhi et al., the GSK3β pathway is assumed to be involved in modulating circadian *CLOCK* gene expression, inflammation, oxidative stress, stress response, neuroplasticity, and monoaminergic neurotransmission (Malhi et al., 2018). All of these items are associated with the phenotype of suicidal ideation and behavior. Taken together, research in this field is scarce and more studies with multidimensional approaches are required. Future promising results could lead to the development of a clinical tool to improve the quality of life of patients with BD.

A basic requirement for future research is the standardized assessment of phenotypes. The bipolar phenotype often appears heterogeneously, and it has a phenotypic overlap with other psychiatric diseases, which leads to challenges in differentiation. Therefore, a standardized assessment should start with a diagnostic classification, e.g., with the Diagnostic and Statistical Manual for Mental Disorders (DSM). In addition, treatment response has to be assessed consistently, especially in pharmacogenetic studies. A milestone in this context is the Alda Scale, a dichotomous and continuous inventory to retrospectively assess lithium response (Manchia et al., 2013). Future studies should use this instrument, and data collectors worldwide should be trained in its use to harmonize phenotypic assessment. Once we acquire proper phenotyping, we can focus on merging information from longitudinal, multidimensional, and “multi-omic” studies. Combining clinical and biological markers could lead to a prediction model, enriched by machine learning approaches. All research efforts in this field aim to improve our knowledge of lithium’s mechanism of action, from the genetic architecture to molecular pathways and the distribution of lithium in the brain. Combining these aspects with clinical stratification of subphenotypes of lithium response is required for precision medicine. One goal is to confirm that lithium is an effective and tolerable treatment option. As an overarching goal, we need to advance precision medicine in psychiatry; to successfully do so, Scott et al. recommend combining clinical assessment and monitoring approaches with applied biological research and analytic approaches (Scott et al., 2018). A breakthrough in patient counseling would be to develop a tool for everyday clinical practice that could predict the efficacy and tolerability of lithium on an individual level. In precision medicine, besides genetic aspects we must also consider lifestyle and environmental factors. Therefore, a broadly diverse multi-method approach to all questions about lithium treatment could lead to a better quality of life for patients and less disability through improved treatment efficacy, fewer side effects, and better adherence. Besides improving patients’ lives, these aspects may help to improve cost efficacy and reduce disability-related costs.

## Conclusion

Several reviews of pharmacogenetic studies on lithium response have been published in recent years (Severino et al., 2013; Geoffroy et al., 2014; Alda, 2015; Pisanu et al., 2016; Budde et al., 2017, 201). Linkage and segregation studies have not been able to detect genetic loci that co-segregate with lithium response, and candidate-gene association studies delivered conflicting results, especially on *GSK3β, BDNF, and SLC6A4*. Replication studies could not confirm the findings of candidate-gene association studies, so their findings should be interpreted with care. GWAS approaches achieved more promising results. However, only limited pharmacogenetic research has been performed on tolerability and anti-suicidal effects. Overall, limiting factors of research to date include insufficient sample sizes and missing replication studies on the one hand and inhomogeneous phenotyping on the other. To move closer to precision medicine, pharmacogenetics has to be enriched by different methodologies. The combination of multi-method and “multi-omic” approaches could facilitate the development of biomarkers for everyday clinical practice. Such biomarkers could advance our objective of being able to offer precision medicine, which is crucial for achieving better treatment options for patients with BD.
