# Supplementary material for: The Genetics of Response to and Side Effects of Lithium Treatment in Bipolar Disorder: Future Research Perspectives
Source: Front Pharmacol. 2021 Mar 25;12:638882. doi: 10.3389/fphar.2021.638882 (PMC8044839; doi:10.3389/fphar.2021.638882)
Supplement: Supplementary file 1 [file table1.docx]

Supplementary Material

**Supplementary Table.** Candidate-gene association studies in lithium response

| **Author/Year** | **Sample size** | **Gene / Locus** | **Results** | **Remarks** |
| --- | --- | --- | --- | --- |
| Ventura et al., 1990 (Ventura et al., 1990) | 36 patients | A2 HLA antigen | A2 HLA antigen more frequently found in non-responders | Serum analyses to identify the presence of antibodies to human leukocyte antigens (HLA) |
| Turecki et al., 1996 (Turecki et al., 1996) | 55 bipolar patients with excellent lithium response and 94 controls | Short arm chromosome 18, including *GNAL* (G Protein Subunit Alpha L) gene | No evidence for association | Case-control analysis of excellent lithium responders versus healthy controls |
| Cavazzoni et al., 1996 (Cavazzoni et al., 1996) | 54 bipolar patients with excellent lithium response and 94 controls | *TH* (Tyrosine hydroxylase) gene | No evidence for association of a tetranucleotide repeat within *TH* gene | Case-control analysis of excellent lithium responders versus healthy controls |
| Turecki et al., 1998 (Turecki et al., 1998) | 136 bipolar patients with excellent lithium response and 163 controls | *PLCG1* (phospholipase C-gamma1) gene | (CA)_n_ repeat associated with bipolar disorder | Case-control analysis of excellent lithium responders versus healthy controls |
| Serretti et al. 1998 (Serretti et al., 1998) | 43 bipolar and 12 major depressive disorder patients treated with lithium | *DRD3 (dopamine receptor D3)* gene | No evidence for association | Case-only analysis of lithium-treated patients |
| Turecki et al., 1999 (Turecki et al., 1999) | 138 bipolar patients with excellent lithium response and 108 controls | *MAOA* (monoamine oxidase A) gene | No evidence for association | Case-control analysis of excellent lithium responders versus healthy controls |
| Serretti et al., 1999 (Serretti et al., 1999b) | 90 bipolar and 18 major depressive disorder patients treated with lithium | *TPH1* (Tryptophan hydroxylase) gene | Marginal association of BfaI polymorphism with pre/on-lithium treatment recurrence index | Case-only analysis of lithium-treated patients |
| Serretti et al., 1999 (Serretti et al., 1999a) | 125 bipolar and 25 major depressive disorder patients treated with lithium | *DRD2* (dopamine receptor D2) gene  *DRD4* (dopamine receptor D4) gene  *GABRA1* (Gamma-Aminobutyric Acid Type A Receptor Alpha1 Subunit) gene | No evidence for association | Case-only analysis of lithium-treated patients |
| Turecki et al., 2000 (Turecki et al., 2000) | 138 bipolar patients with excellent lithium response and 108 controls | 5 loci with polyglutamine (CAG repeat) coding genes | No evidence for association | Case-control analysis of excellent lithium responders versus healthy controls |
| Alda et al., 2000 (Alda et al., 2000) | 138 bipolar patients with excellent lithium response and 108 controls | *CRH* (Corticotropin-Releasing Hormone) gene  *PENK* (Proenkephalin) gene | No evidence for association of the intragenic dinucleotide repeats at these *loci* | Case-control analysis of excellent lithium responders versus healthy controls |
| Duffy et al., 2000 (Duffy et al., 2000) | 138 bipolar patients with excellent lithium response and 108 controls | *GABRA3*  *GABRA5*  *GABRB3*  (Gamma-Aminobutyric Acid Type A Receptor Alpha3-5/Beta3 Subunit) genes | No evidence for association of the intragenic dinucleotide repeats at these *loci* | Case-control analysis of excellent lithium responders versus healthy controls |
| Serretti et al., 2001 (Serretti et al., 2001) | 167 bipolar and 34 major depressive disorder patients treated with lithium | *SLC6A4* (Serotonin transporter) gene | Promoter length polymorphism associated with pre/on-lithium treatment recurrence index | Case-only analysis of lithium-treated patients |
| Serretti et al., 2002 (Serretti et al., 2002) | 160 bipolar and 41 major depressive disorder patients treated with lithium | *COMT* (catechol-O-methyltransferase) gene  *MAOA* (monoamine oxidase A) gene  *GNB3* (G Protein Subunit Beta 3) gene | No evidence for association | Case-only analysis of lithium-treated patients |
| Zill et al., 2003 (Zill et al., 2003) | 149 bipolar patients treated with lithium | *GNAL* (G Protein Subunit Alpha L) gene | No evidence for association of two intronic SNPs | Case-only analysis of lithium-treated patients |
| Washizuka et al., 2003 (Washizuka et al., 2003) | 54 bipolar patients treated with lithium | mtDNA polymorphisms: 5178C>A 10398A>G | 10398A>G associated with lithium response | Case-only analysis of lithium-treated patients |
| Sjøholt et al., 2004 (Sjøholt et al., 2004) | 44 bipolar patients treated with lithium | *IMPA1*  *IMPA2*  (Inositol Monophosphatase 1-2) genes | No evidence for association of 3 *IMPA1* and 10 *IMPA2* polymorphisms | Case-only analysis of lithium-treated patients |
| Serreti et al., 2004 (Serretti et al., 2004) | 83 bipolar patients treated with lithium | *SLC6A4* (Serotonin transporter) gene | No evidence for association | Case-only analysis of lithium-treated patients |
| Dimitrova et al., 2005 (Dimitrova et al., 2005) | 77 bipolar patients treated with lithium.  43 parents–offspring trios | *IMPA2*  (Inositol Monophosphatase 2) gene | No evidence for association in case-only analysis.  TDT shows preferential transmission of rs3786282 and 599+97G>A alleles in good responders trios (N=19) | Case-only and parents–offspring trios analysis of lithium-treated patients |
| Rybakowski et al., 2005 (Rybakowski et al., 2005) | 88 bipolar patients treated with lithium | *BDNF* (Brain-Derived Neurotrophic Factor) gene | Val66Met polymorphism associated with lithium response | Case-only analysis of lithium-treated patients |
| Dmitrzak-Weglarz et al., 2005 (Dmitrzak-Weglarz et al., 2005) | 92 bipolar patients treated with lithium | *HTR2A*  *HTR2C*  (Serotonin receptors 2A/2C) genes | No evidence for association | Case-only analysis of lithium-treated patients |
| Masui et al., 2006 (Masui et al., 2006a) | 66 bipolar patients treated with lithium | *XBP1* (X-Box Binding Protein 1) gene | -116C/G associated with lithium response | Case-only analysis of lithium-treated patients |
| Masoliver et al., 2006 (Masoliver et al., 2006) | 98 bipolar patients treated with lithium | *SLC6A4* (Serotonin transporter) gene | No evidence for association | Case-only analysis of lithium-treated patients |
| Masui et al., 2006 (Masui et al., 2006b) | 161 bipolar patients treated with lithium | *BDNF* (Brain-Derived Neurotrophic Factor) gene | No evidence for association | Case-only analysis of lithium-treated patients |
| Michelon et al., 2006 (Michelon et al., 2006) | 134 bipolar patients treated with lithium | *INPP1* (Inositol Polyphosphate-1-Phosphatase) gene  *SLC6A4* (Serotonin transporter) gene  *BDNF* (Brain-Derived Neurotrophic Factor) gene  *TFAP2B* (Transcription Factor AP-2 Beta) gene  *GSK3ß* (Glycogen Synthase Kinase-3β) gene | No evidence for association | Case-only analysis of lithium-treated patients |
| Szczepankiewicz et al., 2006 (Szczepankiewicz et al., 2006) | 89 bipolar patients treated with lithium | *GSK3ß* (Glycogen synthase kinase-3β) gene | No evidence for association | Case-only analysis of lithium-treated patients |
| Mamdani et al., 2007 (Mamdani et al., 2007) | Approx. 200 bipolar patients treated with lithium | *PREP* (Prolyl Endopeptidase) gene | No evidence for association | Case-only analysis of lithium-treated patients |
| Adli et al., 2007 (Adli et al., 2007) | 81 major depressive disorder patients treated with lithium | *GSK3ß* (Glycogen synthase kinase-3β) gene | −50T/C SNP associated with clinical response to lithium augmentation | Case-only analysis of depressive lithium augmentation-treated patients |
| Masui et al., 2008 (Masui et al., 2008) | 161 bipolar patients treated with lithium | *BCR* (BCR, RhoGEF And GTPase Activating Protein) gene | Asn796Ser polymorphism associated with lithium response | Case-only analysis of lithium-treated patients |
| Mamdani et al., 2008 | Approx. 200 bipolar patients treated with lithium | *CREB1*  *CREB2*  *CREB3*  (CAMP Responsive Element Binding Protein 1/2/3) genes | Two *CREB1* SNPs associated with lithium response | Case-only analysis of lithium-treated patients |
| Silberberg et al., 2008 (Silberberg et al., 2008) | 197 + 161 bipolar patients treated with lithium | *CACNG2* (Calcium Voltage-Gated Channel Auxiliary Subunit Gamma 2) gene | 3 SNPs associated with lithium response | Case-only analysis of lithium-treated patients |
| Squassina et al., 2008 (Squassina et al., 2008) | 155 bipolar patients treated with lithium | *PDLIM5* (PDZ And LIM Domain 5) gene | No evidence for association | Case-only analysis of lithium-treated patients |
| Yun et al., 2008 (Yun et al., 2008) | 45 bipolar patients treated with lithium (n=30) or other mood stabilizers (n=15) | *DTNBP1* (Dystrobrevin Binding Protein 1) gene | No evidence for association with response to mood stabilizers | Case-only analysis of mood stabilizer-treated patients |
| Dmitrzak-Weglarz et al., 2008 (Dmitrzak-Weglarz et al., 2008) | 108 bipolar patients treated with lithium | *BDNF* (Brain-Derived Neurotrophic Factor) gene  *NTRK2* (Neurotrophic Receptor Tyrosine Kinase 2) gene | Val66Met and rs988748 BDNF SNPs associated with lithium response | Case-only analysis of lithium-treated patients |
| Rybakowski et al., 2009 (Rybakowski et al., 2009) | 92 bipolar patients treated with lithium | *DRD1* (dopamine receptor D1) gene | -48A/G polymorphism associated with lithium response | Case-only analysis of lithium-treated patients |
| Szczepankiewicz et al., 2009 (Szczepankiewicz et al., 2009b) | 101 bipolar patients treated with lithium | *FYN* (FYN Proto-Oncogene, Src Family Tyrosine Kinase) gene | No evidence for association | Case-only analysis of lithium-treated patients |
| Szczepankiewicz et al., 2009 (Szczepankiewicz et al., 2009a) | 105 bipolar patients treated with lithium | *GRIN2B* (Glutamate Ionotropic Receptor NMDA Type Subunit 2B) gene | No evidence for association | Case-only analysis of lithium-treated patients |
| Manchia et al., 2009 (Manchia et al., 2009a) | 155 bipolar patients treated with lithium | *DRD1*  DRD2  DRD3 (dopamine receptor D1/2/3) genes  *SLC6A3* (Dopamine transporter) gene  *SLC6A4* (Serotonin transporter) gene  *HTR2A*  (Serotonin receptors 2A) gene | No evidence for association | Case-only analysis of lithium-treated patients |
| Manchia et al., 2009 (Manchia et al., 2009b) | 199 bipolar patients treated with lithium | *DGKH* (Diacylglycerol Kinase Eta) gene  *NR1D1* (Nuclear Receptor Subfamily 1 Group D Member 1) gene | No evidence for association | Case-only analysis of lithium-treated patients |
| Campos de Sousa et al., 2010 (Campos-de-Sousa et al., 2010) | 170 bipolar patients treated with lithium | *NR1D1* (Nuclear Receptor Subfamily 1 Group D Member 1) gene | rs231433 associated with lithium response | Case-only analysis of lithium-treated patients |
| Drago et al., 2010 (Drago et al., 2010) | 83 bipolar patients treated with lithium | *BDNF* (Brain-Derived Neurotrophic Factor) gene | No evidence for association | Case-only analysis of lithium-treated patients |
| Rybakowski et al., 2011 (Rybakowski et al., 2011) | 109 bipolar patients treated with lithium | *MMP9* (Matrix Metallopeptidase 9) gene | No evidence for association | Case-only analysis of lithium-treated patients |
| McCarthy et al., 2011 (McCarthy et al., 2011) | 282 bipolar patients treated with lithium | *NR1D1* (Nuclear Receptor Subfamily 1 Group D Member 1)  *ARNTL* (Aryl Hydrocarbon Receptor Nuclear Translocator Like)  *CLOCK* (Clock Circadian Regulator)  *CRY1* (Cryptochrome Circadian Regulator 1)  *GSK3ß* (Glycogen synthase kinase-3β)  *PER2* (Period Circadian Regulator 2)  *PER3* (Period Circadian Regulator 3)  genes | rs8192440 (*CRY1*) and rs2071427 (*NR1D1*) associated with lithium response.  Combined effect of rs2071427 (*NR1D1*) and rs6438552 (*GSK3ß*) on lithium response | Case-only analysis of lithium-treated patients |
| Szczepankiewicz et al., 2011 (Szczepankiewicz et al., 2011) | 115 bipolar patients treated with lithium | *NR3C1* (Glucocorticoid receptor) gene | rs41423247 associated with lithium response | Case-only analysis of lithium-treated patients |
| Squassina et al., 2011 (Squassina et al., 2011) | 52 bipolar patients treated with lithium | *ACCN1* gene (Amiloride-sensitive Cation Channel 1, Neuronal) or new name: *ASIC2* (Acid-sensing ion channel subunit 2) / chromosome 17q12 | Robust association between rs11869731 and lithium response | Case-only analysis of lithium-treated patients (full-responders and non-responders) |
| Numajiri et al., 2012 (Numajiri et al., 2012) | 29 bipolar patients treated with lithium | *GSK3β* (Glycogen synthase kinase-3β) gene | *GSK3ß* -50T/C may be linked with the effect of lithium treatment, and *GSK3ß* -1727A/T has no significant relationship | Case-only analysis of lithium-treated patients |
| Rybakowski et al., 2012 (Rybakowski et al., 2012) | 101 bipolar patients treated with lithium | *5HTT, DRD1, COMT, BDNF, FYN, 5HT2A, 5HT2C, DRD2, DRD3, DRD4, GSK3, NTRK2, GRIN2B,* and *MMP-9* genes | Association with lithium prophylactic efficacy was found for *5HTT* (ins/del 44 pz), *DRD1* (-48 A/G), *COMT* (Val108Met), *BDNF* (Val66Met) and *FYN* (37 T/C); No association for *5HT2A*, *5HT2C*, *DRD2*, *DRD3*, *DRD4*, *GSK3*, *NTRK2*, *GRIN2B* and *MMP-9* | Case-only analysis of lithium-treated patients (excellent-responders, partial-responders, and non-responders) |
| [Benedetti](https://pubmed.ncbi.nlm.nih.gov/?term=Benedetti+F&cauthor_id=22119086) et al., 2012 (Benedetti et al., 2012) | 122 patients affected by a major depressive episode in the course of bipolar treated with lithium | *GSK3β* (Glycogen synthase kinase-3β); *5-HTTLPR* (Serotonin transporter) genes | Among rs334558 T/T homozygotes (*GSK3β*), the best antidepressant response was associated with *5-HTTLPR* l/l homozygosity; among the rs334558 C carriers (*GSK3β*), the *5-HTTLPR* s/s showed the best response to treatment | Case-only analysis of lithium-treated patients |
| Wang et al., 2012 (Wang et al., 2012) | 342 bipolar I and II patients treated with lithium or valproate and 386 healthy controls | *BDNF* (brain-derived neurotrophic factor) gene | Association of rs6265 (Val66Met polymorphism) with lithium response in both BPD-I and BPD-II patients | Case-control analysis of lithium or valproate responder treated patients versus healthy controls |
| Pisanu et al., 2013 (Pisanu et al., 2013) | 204 bipolar patients treated with lithium and 422 healthy controls | *NAPE*-*PLD* (N-acyl phosphatidylethanolamine phospholipase D), *CNR1* (cannabinoid receptor1), and *FAAH* (fatty acid amide hydrolase) genes | None of the SNPs of *NAPE-PLD*, *CNR1*, or *FAAH* showed nominal association with lithium response | Case-control analysis of lithium-treated patients versus healthy controls |
| Tharoor e al., 2013 (Tharoor et al., 2013) | 122 bipolar patients treated with lithium and 124 healthy controls | Triallelic 5-hydroxy tryptamine transporter linked promoter region (*5-HTTLPR*) and variable number tandem repeats in the serotonin transporter intron 2 (STin2) | Significant association of haplotype consisting of the S allele of *5-HTTLPR* and 10 repeat allele of STin2 (STin2.10) with lithium response | Case-control analysis of lithium-treated patients (good responders, non-responders, and partial responders) versus healthy controls |
| Wang et al., 2013 (Wang et al., 2013, 2) | 284 bipolar I patients treated with lithium or valproate and 295 healthy controls | *NTRK2* (Neurotrophic tyrosine kinase receptor type 2) gene | Significant allelic association between rs2769605 and response to lithium or valproate; No significant association between rs1387923 and rs1565445 and response to lithium or valproate | Case-control analysis of excellent lithium or valproate responders versus healthy controls |
| Lin et al., 2013 (Lin et al., 2013) | 83 bipolar I patients treated with lithium and 131 healthy controls | *GSK3β* (Glycogen synthase kinase-3β) gene/chromosome 3q13 | Genotype TT at rs334558 had a poorer response to lithium treatment than genotypes CC and CT | Case-control analysis of lithium-treated patients and non-lithium-treated patients versus healthy controls |
| Iwahashi et al., 2014 (Iwahashi et al., 2014) | 42 bipolar patients treated with lithium | *GSK3β* (Glycogen synthase kinase–3β) gene | No significant difference in genotypic or allelic frequencies of single SNPs -50T/C (rs334558) and -1727A/T (rs3755557) between lithium responders and non-responders; *GSK3ß* haplotype T-A was associated with a higher lithium response, and haplotype C-A was associated with a lower lithium response | Case-only analysis of lithium-treated patients (responders and non-responders) |
| Wang et al., 2014 (Wang et al., 2014) | 280 bipolar I patients treated with lithium and valproate and 288 healthy controls | *MIR206* (*miRNA*-206) and *BDNF* (brain-derived neurotrophic factor) genes | No association was observed in the individual polymorphism, *MIR206* rs16882131 and *BDNF* rs6265, with lithium and valproate response; Individuals with *MIR206* T/T + TC and *BDNF* A/A genotypes had a significantly lower mean treatment score than those with *MIR206* CC and *BDNF* A/A + A/G as well as *MIR206* CC and *BDNF* G/G genotypes | Case-control analysis of lithium and valproate treated bipolar patients versus healthy controls |
| Chen et al., 2014 (Chen et al., 2014) | 294, 100, 24, 94 and 94 bipolar patients treated with lithium | *GADL1* (Glutamate decarboxylase-like protein 1) gene / chromosome 3p24 | rs17026688 and rs17026651 and *GADL1* IVS8+48delG are associated with response to lithium and can be useful biomarkers in predicting the response in Asian ancestry | Case-only analysis of lithium-treated patients (excellent lithium responders, poor responders, and non-responders) |
| Rybakowski et al., 2014 (Rybakowski et al., 2014) | 115 bipolar patients with lithium prophylaxis | *CLOCK* (Circadian Locomotor Output Cycle Kaput), *ARNTL* (Aryl hydrocarbon Receptor Nuclear Translocator-Like), *TIMELESS* (Timeless circadian clock), *PER3* (Period circadian clock 3) genes | Association with the degree of lithium prophylaxis response was found with eight polymorphisms of the *ARNTL* gene (rs4146388, rs10766075, rs7396943, rs11824092, rs11600996, rs11022780, rs7107287, and rs1982350) and two SNPs of the *TIM* gene (rs10876890 and rs2279665); No association was found with any of the polymorphisms of the *CLOCK* and *PER3* genes | Case-only analysis of lithium-treated patients |
| Mitjans et al., 2015 (Mitjans et al., 2015, 3) | 131 bipolar patients treated with lithium | *IMPA2*, *INNP1*, *GSK3ß*, and *GRIK2* genes | Significant association for rs669838-C (I*MPA2*), rs909270-G (*INPP1*), rs11921360-A (*GSK3ß*) and rs28522620 (*GRIK2*) with lithium nonresponse; Significant association for the haplotypes rs3791809-rs4853694-rs909270 (*INPP1*) and rs1732170-rs11921360-rs334558 (*GSK3ß)* and lithium response | Case-only analysis of lithium-treated patients |
| Kotambail et al., 2015 (Kotambail et al., 2015) | 151 patients treated with lithium | *GADL1* (glutamate decarboxylase-like protein 1) gene | No evidence for association of  rs17026688 and rs17026651 | Case-only analysis of lithium-treated patients (good responders and poor responders) |
| Geoffroy et al., 2016 (Geoffroy et al., 2016) | 223 bipolar patients treated with lithium | *RORA* (RAR-related orphan receptor-a) and *PPARGC1A* or *PGC*-*1α* (Peroxisome Proliferator-Activated Receptor Gamma, Coactivator 1 Alpha) genes | rs17204573 of RORA and rs9291455 and rs10517026 of PPARGC1A showed associations with lithium response | Case-only analysis of lithium-treated patients |
| Song et al., 2016 (Song et al., 2016) | 323 bipolar patients treated with lithium and 6684 controls | *SESTD1* (SEC14 and spectrin domains 1) gene/ chromosome 2q31.2 | rs116323614 located in an intron of SESTD1 is associated with a lithium-responsive subtype of bipolar disorder | Case-control analysis of lithium-responder treated patients versus controls |
| [Hou et](https://www.ncbi.nlm.nih.gov/pubmed/?term=Hou%20L%5BAuthor%5D&cauthor=true&cauthor_uid=26806518) al., 2016 (Hou et al., 2016) | 2563 bipolar patients treated with lithium | *AL157359*.4 and *AL157359*.3 genes (lncRNA genes)/ Chromosome 21 | Robust association of rs74795342 and rs75222709 of the AL157359.3 gene and rs79663003 and rs78015114 that lie between these two lncRNA genes with the continuous lithium response | Case-only analysis of lithium-treated patients |
| Benedetti et al., 2018 (Benedetti et al., 2018) | 158 patients affected by a major depressive episode in the course of bipolar I treated with lithium | Homer 1 gene / chr. 5q14.2 | rs7713917 influenced long term effects of lithium on white matter structure; Lithium treatment increased axial diffusivity more in AA patients than in G*carriers | Case-only analysis of lithium-treated patients |
| Szczepankiewicz et al., 2018 (Szczepankiewicz et al., 2018) | 93 bipolar patients treated with lithium | *CRHR1* (Corticotropin-releasing hormone receptor 1), *AVPR1b* (Arginine vasopressin receptor 1B), *FKBP* *5* (FK506 binding protein 5), *FKBP4*, *BAG1* (BCL2-associated athanogene 1), *STIP1* (Stress-induced phosphoprotein 1), *GLCC1* (Glucocorticoid-induced transcript 1), *DUSP1* (Dual specificity phosphatase 1), *SRSF* 3 (Serine and arginine-rich splicing factor 3), *SRSF9*, *SRSF5*, and *ACP1* (Acid phosphatase 1) genes | Significant association between three *FKBP5* polymorphisms (rs1360780, rs7748266, and rs9296158), one *ACP1* variant (rs300774) and one *GLCC1* variant (rs37972) and the degree of lithium response, No association was found between the other analyzed gene polymorphisms within *CRHR1, AVPR1β, FKBP4, BAG1, STIP1, DUSP1, SRSF3*, and *SRSF5* and the lithium response | Case-only analysis of lithium-treated patients |
| Pisanu et al., 2018 (Pisanu et al., 2018) | 205 bipolar patients treated with lithium | *ZNF493* (Zinc finger protein 493) gene / Chr 19p12 | rs12975981 was significantly associated with lithium response | Case-only analysis of lithium-treated patients (responder and non-responders) |
| Reinbold et al., 2018 (Reinbold et al., 2018) | 2563 bipolar patients treated with lithium | *micro*-*RNA* genes | No association between miRNAs and treatment response to lithium in BD in either of the tested conditions withstood multiple testing correction | Case-only analysis of lithium-treated patients |
| Amare et al., 2018 (Amare et al., 2018) | 2586 bipolar patients treated with lithium | Genes*:*  *MEF2C/ Chr 5;*  *CMAHP/ Chr 6;*  *HCG4/ Chr 6;*  *EPHX2/ Chr 8;*  *GRAMD1B/ Chr 11;*  *MYO1H/ Chr 12;*  *ADAMTSL3/ Chr 15;*  *ERCC4/ Chr 16;*  *FAM178B/ Chr 2;*  *ZNF804A/ Chr 2;*  *MAIP1/ Chr 2;*  *CCNH/ Chr 5;*  *HCG4/ Chr 6;*  *HLA-DMA/ Chr 6;*  *ADCY1/ Chr 7;*  *FAM177A1/ Chr 14* | According to order of the genes: rs324899; rs6942227; rs142425863; rs59724122; rs61123830; rs7959663; rs66486766; rs7405404; rs6728642; rs62200793; rs7588746; rs3919583; rs144373461; rs209474; rs1521470; rs79403677, an inverse association between genetic loading for schizophrenia risk variants and response to lithium in patients with bipolar affective disorder | Case-only analysis of lithium-treated patients and cross-trait meta-analysis of GWAS on lithium treatment response in patients with BPAD and GWAS on SCZ (36,989 patients with SCZ) |
| Miranda et al., 2019 (Miranda et al., 2019) | 286 and 68 bipolar patients treated with lithium (Retrospective and Prospective studies, respectively) | *CACNG2* gene | Top retrospective SNP (rs140040) and top prospective SNP (rs2283967) were associated with lithium response | Case-only analysis of lithium-treated patients (good responders and poor responders) |
| Pisanu et al., 2019 (Pisanu et al., 2019) | 24 bipolar patients treated with lithium | *miR*-*320* and *miR*-*155* genes | miR-320a and miR-155-3p, as well as three of their targeted genes (*CAPNS1* (Calpain Small Subunit 1) and *RGS16* (Regulator of G Protein Signaling 16) for miR-320, SP4 (Sp4 Transcription Factor) for *miR-155*-3p), might be involved in modulating lithium response | Case-only analysis of lithium-treated patients (excellent responders and non-responders) |
| Jacobs et al., 2020 (Jacobs et al., 2020) | 5 members of a family of lithium response discordant bipolar monozygotic twins | *NF1* (Neurofibromin type 1) gene / 17q11.2 | Association of a non-synonymous single-nucleotide variant (nsSNP) causing p.Asp1067-Val and a frameshift mutation due to deletion (CAGAG/Del) (p.Asp1067fs) with poor response to lithium | Case-control analysis of lithium-treated patients versus healthy controls |

**References**

Adli, M., Hollinde, D. L., Stamm, T., Wiethoff, K., Tsahuridu, M., Kirchheiner, J., et al. (2007). Response to lithium augmentation in depression is associated with the glycogen synthase kinase 3-beta -50T/C single nucleotide polymorphism. *Biol. Psychiatry* 62, 1295–1302. doi:10.1016/j.biopsych.2007.03.023.

Alda, M., Turecki, G., Grof, P., Cavazzoni, P., Duffy, A., Grof, E., et al. (2000). Association and linkage studies of CRH and PENK genes in bipolar disorder: a collaborative IGSLI study. *Am. J. Med. Genet.* 96, 178–181.

Amare, A. T., Schubert, K. O., Hou, L., Clark, S. R., Papiol, S., Heilbronner, U., et al. (2018). Association of Polygenic Score for Schizophrenia and HLA Antigen and Inflammation Genes With Response to Lithium in Bipolar Affective Disorder: A Genome-Wide Association Study. *JAMA Psychiatry* 75, 65–74. doi:10.1001/jamapsychiatry.2017.3433.

Benedetti, F., Dallaspezia, S., Lorenzi, C., Pirovano, A., Radaelli, D., Locatelli, C., et al. (2012). Gene-gene interaction of glycogen synthase kinase 3-beta and serotonin transporter on human antidepressant response to sleep deprivation. *J Affect Disord* 136, 514–9. doi:10.1016/j.jad.2011.10.039.

Benedetti, F., Poletti, S., Locatelli, C., Mazza, E., Lorenzi, C., Vitali, A., et al. (2018). A Homer 1 gene variant influences brain structure and function, lithium effects on white matter, and antidepressant response in bipolar disorder: A multimodal genetic imaging study. *Prog Neuropsychopharmacol Biol Psychiatry* 81, 88–95. doi:10.1016/j.pnpbp.2017.10.011.

Campos-de-Sousa, S., Guindalini, C., Tondo, L., Munro, J., Osborne, S., Floris, G., et al. (2010). Nuclear receptor rev-erb-{alpha} circadian gene variants and lithium carbonate prophylaxis in bipolar affective disorder. *J. Biol. Rhythms* 25, 132–137. doi:10.1177/0748730410362713.

Cavazzoni, P., Alda, M., Turecki, G., Rouleau, G., Grof, E., Martin, R., et al. (1996). Lithium-responsive affective disorders: no association with the tyrosine hydroxylase gene. *Psychiatry Res.* 64, 91–96.

Chen, C. H., Lee, C. S., Lee, M. T., Ouyang, W. C., Chen, C. C., Chong, M. Y., et al. (2014). Variant GADL1 and response to lithium therapy in bipolar I disorder. *N Engl J Med* 370, 119–28. doi:10.1056/NEJMoa1212444.

Dimitrova, A., Milanova, V., Krastev, S., Nikolov, I., Toncheva, D., Owen, M. J., et al. (2005). Association study of myo-inositol monophosphatase 2 (IMPA2) polymorphisms with bipolar affective disorder and response to lithium treatment. *Pharmacogenomics J.* 5, 35–41. doi:10.1038/sj.tpj.6500273.

Dmitrzak-Weglarz, M., Rybakowski, J. K., Suwalska, A., Skibinska, M., Leszczynska-Rodziewicz, A., Szczepankiewicz, A., et al. (2008). Association studies of the BDNF and the NTRK2 gene polymorphisms with prophylactic lithium response in bipolar patients. *Pharmacogenomics* 9, 1595–1603. doi:10.2217/14622416.9.11.1595.

Dmitrzak-Weglarz, M., Rybakowski, J. K., Suwalska, A., Słopień, A., Czerski, P. M., Leszczyńska-Rodziewicz, A., et al. (2005). Association studies of 5-HT2A and 5-HT2C serotonin receptor gene polymorphisms with prophylactic lithium response in bipolar patients. *Pharmacol. Rep. PR* 57, 761–765.

Drago, A., Serretti, A., Smith, R., Huezo-Diaz, P., Malitas, P., Albani, D., et al. (2010). No association between genetic markers in BDNF gene and lithium prophylaxis in a Greek sample. *Int. J. Psychiatry Clin. Pract.* 14, 154–157. doi:10.3109/13651501003706717.

Duffy, A., Turecki, G., Grof, P., Cavazzoni, P., Grof, E., Joober, R., et al. (2000). Association and linkage studies of candidate genes involved in GABAergic neurotransmission in lithium-responsive bipolar disorder. *J. Psychiatry Neurosci. JPN* 25, 353–358.

Geoffroy, P. A., Etain, B., Lajnef, M., Zerdazi, E. H., Brichant-Petitjean, C., Heilbronner, U., et al. (2016). Circadian genes and lithium response in bipolar disorders: associations with PPARGC1A (PGC-1alpha) and RORA. *Genes Brain Behav* 15, 660–8. doi:10.1111/gbb.12306.

Hou, L., Heilbronner, U., Degenhardt, F., Adli, M., Akiyama, K., Akula, N., et al. (2016). Genetic variants associated with response to lithium treatment in bipolar disorder: a genome-wide association study. *Lancet* 387, 1085–1093. doi:10.1016/s0140-6736(16)00143-4.

Iwahashi, K., Nishizawa, D., Narita, S., Numajiri, M., Murayama, O., Yoshihara, E., et al. (2014). Haplotype analysis of GSK-3beta gene polymorphisms in bipolar disorder lithium responders and non-responders. *Clin Neuropharmacol* 37, 108–10. doi:10.1097/wnf.0000000000000039.

Jacobs, A., Hagin, M., Shugol, M., Shomron, N., Pillar, N., Fananas, L., et al. (2020). The black sheep of the family- whole-exome sequencing in family of lithium response discordant bipolar monozygotic twins. *Eur Neuropsychopharmacol*. doi:10.1016/j.euroneuro.2020.03.009.

Kotambail, A., Mathur, A., Bhat, S. M., Rai, P. S., Sharma, P. S., and Satyamoorthy, K. (2015). GADL1 gene polymorphisms and lithium response in bipolar I disorder: lack of association from an Indian population. *Psychiatr Genet* 25, 39–40. doi:10.1097/ypg.0000000000000066.

Lin, Y. F., Huang, M. C., and Liu, H. C. (2013). Glycogen synthase kinase 3beta gene polymorphisms may be associated with bipolar I disorder and the therapeutic response to lithium. *J Affect Disord* 147, 401–6. doi:10.1016/j.jad.2012.08.025.

Mamdani, F., Sequeira, A., Alda, M., Grof, P., Rouleau, G., and Turecki, G. (2007). No association between the PREP gene and lithium responsive bipolar disorder. *BMC Psychiatry* 7, 9. doi:10.1186/1471-244X-7-9.

Manchia, M., Congiu, D., Squassina, A., Lampus, S., Ardau, R., Chillotti, C., et al. (2009a). No association between lithium full responders and the DRD1, DRD2, DRD3, DAT1, 5-HTTLPR and HTR2A genes in a Sardinian sample. *Psychiatry Res.* 169, 164–166. doi:10.1016/j.psychres.2008.12.004.

Manchia, M., Squassina, A., Congiu, D., Chillotti, C., Ardau, R., Severino, G., et al. (2009b). Interacting genes in lithium prophylaxis: preliminary results of an exploratory analysis on the role of DGKH and NR1D1 gene polymorphisms in 199 Sardinian bipolar patients. *Neurosci. Lett.* 467, 67–71. doi:10.1016/j.neulet.2009.10.003.

Masoliver, E., Menoyo, A., Pérez, V., Volpini, V., Rio, E. D., Pérez, J., et al. (2006). Serotonin transporter linked promoter (polymorphism) in the serotonin transporter gene may be associated with antidepressant-induced mania in bipolar disorder. *Psychiatr. Genet.* 16, 25–29.

Masui, T., Hashimoto, R., Kusumi, I., Suzuki, K., Tanaka, T., Nakagawa, S., et al. (2006a). A possible association between the -116C/G single nucleotide polymorphism of the XBP1 gene and lithium prophylaxis in bipolar disorder. *Int. J. Neuropsychopharmacol.* 9, 83–88. doi:10.1017/S1461145705005523.

Masui, T., Hashimoto, R., Kusumi, I., Suzuki, K., Tanaka, T., Nakagawa, S., et al. (2006b). Lithium response and Val66Met polymorphism of the brain-derived neurotrophic factor gene in Japanese patients with bipolar disorder. *Psychiatr. Genet.* 16, 49–50. doi:10.1097/01.ypg.0000180680.72922.57.

Masui, T., Hashimoto, R., Kusumi, I., Suzuki, K., Tanaka, T., Nakagawa, S., et al. (2008). A possible association between missense polymorphism of the breakpoint cluster region gene and lithium prophylaxis in bipolar disorder. *Prog. Neuropsychopharmacol. Biol. Psychiatry* 32, 204–208. doi:10.1016/j.pnpbp.2007.08.010.

McCarthy, M. J., Nievergelt, C. M., Shekhtman, T., Kripke, D. F., Welsh, D. K., and Kelsoe, J. R. (2011). Functional genetic variation in the Rev-Erbα pathway and lithium response in the treatment of bipolar disorder. *Genes Brain Behav.* 10, 852–861. doi:10.1111/j.1601-183X.2011.00725.x.

Michelon, L., Meira-Lima, I., Cordeiro, Q., Miguita, K., Breen, G., Collier, D., et al. (2006). Association study of the INPP1, 5HTT, BDNF, AP-2beta and GSK-3beta GENE variants and restrospectively scored response to lithium prophylaxis in bipolar disorder. *Neurosci. Lett.* 403, 288–293. doi:10.1016/j.neulet.2006.05.001.

Miranda, A., Shekhtman, T., McCarthy, M., DeModena, A., Leckband, S. G., and Kelsoe, J. R. (2019). Study of 45 candidate genes suggests CACNG2 may be associated with lithium response in bipolar disorder. *J Affect Disord* 248, 175–179. doi:10.1016/j.jad.2019.01.010.

Mitjans, M., Arias, B., Jimenez, E., Goikolea, J. M., Saiz, P. A., Garcia-Portilla, M. P., et al. (2015). Exploring Genetic Variability at PI, GSK3, HPA, and Glutamatergic Pathways in Lithium Response: Association With IMPA2, INPP1, and GSK3B Genes. *J Clin Psychopharmacol* 35, 600–4. doi:10.1097/jcp.0000000000000382.

Numajiri, M., Aoki, J., Iwahashi, K., Fukamauchi, F., Enomoto, M., Yoshihara, E., et al. (2012). [association between lithium sensitivity and GSK3beta gene polymorphisms in bipolar disorder]. *Nihon Shinkei Seishin Yakurigaku Zasshi* 32, 161–3.

Pisanu, C., Congiu, D., Costa, M., Chillotti, C., Ardau, R., Severino, G., et al. (2018). Convergent analysis of genome-wide genotyping and transcriptomic data suggests association of zinc finger genes with lithium response in bipolar disorder. *Am J Med Genet B Neuropsychiatr Genet* 177, 658–664. doi:10.1002/ajmg.b.32663.

Pisanu, C., Congiu, D., Costa, M., Sestu, M., Chillotti, C., Ardau, R., et al. (2013). No association of endocannabinoid genes with bipolar disorder or lithium response in a Sardinian sample. *Psychiatry Res* 210, 887–90. doi:10.1016/j.psychres.2013.09.025.

Pisanu, C., Merkouri Papadima, E., Melis, C., Congiu, D., Loizedda, A., Orru, N., et al. (2019). Whole Genome Expression Analyses of miRNAs and mRNAs Suggest the Involvement of miR-320a and miR-155-3p and their Targeted Genes in Lithium Response in Bipolar Disorder. *Int J Mol Sci* 20. doi:10.3390/ijms20236040.

Reinbold, C. S., Forstner, A. J., Hecker, J., Fullerton, J. M., Hoffmann, P., Hou, L., et al. (2018). Analysis of the Influence of microRNAs in Lithium Response in Bipolar Disorder. *Front Psychiatry* 9, 207. doi:10.3389/fpsyt.2018.00207.

Rybakowski, J. K., Czerski, P., Dmitrzak-Weglarz, M., Kliwicki, S., Leszczynska-Rodziewicz, A., Permoda-Osip, A., et al. (2012). Clinical and pathogenic aspects of candidate genes for lithium prophylactic efficacy. *J Psychopharmacol* 26, 368–73. doi:10.1177/0269881111415736.

Rybakowski, J. K., Dmitrzak-Weglar, M., Kliwicki, S., and Hauser, J. (2014). Polymorphism of circadian clock genes and prophylactic lithium response. *Bipolar Disord* 16, 151–8. doi:10.1111/bdi.12136.

Rybakowski, J. K., Dmitrzak-Weglarz, M., Suwalska, A., Leszczynska-Rodziewicz, A., and Hauser, J. (2009). Dopamine D1 receptor gene polymorphism is associated with prophylactic lithium response in bipolar disorder. *Pharmacopsychiatry* 42, 20–22. doi:10.1055/s-0028-1085441.

Rybakowski, J. K., Skibinska, M., Suwalska, A., Leszczynska-Rodziewicz, A., Kaczmarek, L., and Hauser, J. (2011). Functional polymorphism of matrix metalloproteinase-9 (MMP-9) gene and response to lithium prophylaxis in bipolar patients. *Hum. Psychopharmacol.* 26, 168–171. doi:10.1002/hup.1182.

Rybakowski, J. K., Suwalska, A., Skibinska, M., Szczepankiewicz, A., Leszczynska-Rodziewicz, A., Permoda, A., et al. (2005). Prophylactic lithium response and polymorphism of the brain-derived neurotrophic factor gene. *Pharmacopsychiatry* 38, 166–170. doi:10.1055/s-2005-871239.

Serretti, A., Lilli, R., Lorenzi, C., Franchini, L., Di Bella, D., Catalano, M., et al. (1999a). Dopamine receptor D2 and D4 genes, GABA(A) alpha-1 subunit genes and response to lithium prophylaxis in mood disorders. *Psychiatry Res.* 87, 7–19.

Serretti, A., Lilli, R., Lorenzi, C., Franchini, L., and Smeraldi, E. (1998). Dopamine receptor D3 gene and response to lithium prophylaxis in mood disorders. *Int. J. Neuropsychopharmacol.* 1, 125–129. doi:10.1017/S1461145798001230.

Serretti, A., Lilli, R., Lorenzi, C., Gasperini, M., and Smeraldi, E. (1999b). Tryptophan hydroxylase gene and response to lithium prophylaxis in mood disorders. *J. Psychiatr. Res.* 33, 371–377.

Serretti, A., Lilli, R., Mandelli, L., Lorenzi, C., and Smeraldi, E. (2001). Serotonin transporter gene associated with lithium prophylaxis in mood disorders. *Pharmacogenomics J.* 1, 71–77.

Serretti, A., Lorenzi, C., Lilli, R., Mandelli, L., Pirovano, A., and Smeraldi, E. (2002). Pharmacogenetics of lithium prophylaxis in mood disorders: analysis of COMT, MAO-A, and Gbeta3 variants. *Am. J. Med. Genet.* 114, 370–379. doi:10.1002/ajmg.10357.

Serretti, A., Malitas, P. N., Mandelli, L., Lorenzi, C., Ploia, C., Alevizos, B., et al. (2004). Further evidence for a possible association between serotonin transporter gene and lithium prophylaxis in mood disorders. *Pharmacogenomics J.* 4, 267–273. doi:10.1038/sj.tpj.6500252.

Silberberg, G., Levit, A., Collier, D., St Clair, D., Munro, J., Kerwin, R. W., et al. (2008). Stargazin involvement with bipolar disorder and response to lithium treatment. *Pharmacogenet. Genomics* 18, 403–412. doi:10.1097/FPC.0b013e3282f974ca.

Sjøholt, G., Ebstein, R. P., Lie, R. T., Berle, J. Ø., Mallet, J., Deleuze, J. F., et al. (2004). Examination of IMPA1 and IMPA2 genes in manic-depressive patients: association between IMPA2 promoter polymorphisms and bipolar disorder. *Mol. Psychiatry* 9, 621–629. doi:10.1038/sj.mp.4001460.

Song, J., Bergen, S. E., Di Florio, A., Karlsson, R., Charney, A., Ruderfer, D. M., et al. (2016). Genome-wide association study identifies SESTD1 as a novel risk gene for lithium-responsive bipolar disorder. *Mol Psychiatry* 21, 1290–7. doi:10.1038/mp.2015.165.

Squassina, A., Congiu, D., Manconi, F., Manchia, M., Chillotti, C., Lampus, S., et al. (2008). The PDLIM5 gene and lithium prophylaxis: an association and gene expression analysis in Sardinian patients with bipolar disorder. *Pharmacol. Res.* 57, 369–373. doi:10.1016/j.phrs.2008.03.011.

Squassina, A., Manchia, M., Borg, J., Congiu, D., Costa, M., Georgitsi, M., et al. (2011). Evidence for association of an ACCN1 gene variant with response to lithium treatment in Sardinian patients with bipolar disorder. *Pharmacogenomics* 12, 1559–69. doi:10.2217/pgs.11.102.

Szczepankiewicz, A., Narozna, B., Rybakowski, J. K., Kliwicki, S., Czerski, P., Dmitrzak-Weglarz, M., et al. (2018). Genes involved in stress response influence lithium efficacy in bipolar patients. *Bipolar Disord* 20, 753–760. doi:10.1111/bdi.12639.

Szczepankiewicz, A., Rybakowski, J. K., Suwalska, A., and Hauser, J. (2011). Glucocorticoid receptor polymorphism is associated with lithium response in bipolar patients. *Neuro Endocrinol. Lett.* 32, 545–551.

Szczepankiewicz, A., Rybakowski, J. K., Suwalska, A., Skibinska, M., Leszczynska-Rodziewicz, A., Dmitrzak-Weglarz, M., et al. (2006). Association study of the glycogen synthase kinase-3beta gene polymorphism with prophylactic lithium response in bipolar patients. *World J. Biol. Psychiatry Off. J. World Fed. Soc. Biol. Psychiatry* 7, 158–161. doi:10.1080/15622970600554711.

Szczepankiewicz, A., Skibińska, M., Suwalska, A., Hauser, J., and Rybakowski, J. K. (2009a). No association of three GRIN2B polymorphisms with lithium response in bipolar patients. *Pharmacol. Rep. PR* 61, 448–452.

Szczepankiewicz, A., Skibinska, M., Suwalska, A., Hauser, J., and Rybakowski, J. K. (2009b). The association study of three FYN polymorphisms with prophylactic lithium response in bipolar patients. *Hum. Psychopharmacol.* 24, 287–291. doi:10.1002/hup.1018.

Tharoor, H., Kotambail, A., Jain, S., Sharma, P. S., and Satyamoorthy, K. (2013). Study of the association of serotonin transporter triallelic 5-HTTLPR and STin2 VNTR polymorphisms with lithium prophylaxis response in bipolar disorder. *Psychiatr Genet* 23, 77–81. doi:10.1097/YPG.0b013e32835d6fad.

Turecki, G., Alda, M., Grof, P., Joober, R., Lafrenière, R., Cavazzoni, P., et al. (2000). Polyglutamine coding genes in bipolar disorder: lack of association with selected candidate loci. *J. Affect. Disord.* 58, 63–68.

Turecki, G., Alda, M., Grof, P., Martin, R., Cavazzoni, P. A., Duffy, A., et al. (1996). No association between chromosome-18 markers and lithium-responsive affective disorders. *Psychiatry Res.* 63, 17–23.

Turecki, G., Grof, P., Cavazzoni, P., Duffy, A., Grof, E., Ahrens, B., et al. (1998). Evidence for a role of phospholipase C-gamma1 in the pathogenesis of bipolar disorder. *Mol. Psychiatry* 3, 534–538.

Turecki, G., Grof, P., Cavazzoni, P., Duffy, A., Grof, E., Ahrens, B., et al. (1999). MAOA: association and linkage studies with lithium responsive bipolar disorder. *Psychiatr. Genet.* 9, 13–16.

Ventura, T., Lobo, A., and Marco, J. C. (1990). [HLA antigens in bipolar affective patients]. *Actas Luso. Esp. Neurol. Psiquiatr. Cienc. Afines* 18, 339–343.

Wang, Z., Fan, J., Gao, K., Li, Z., Yi, Z., Wang, L., et al. (2013). Neurotrophic tyrosine kinase receptor type 2 (NTRK2) gene associated with treatment response to mood stabilizers in patients with bipolar I disorder. *J Mol Neurosci* 50, 305–10. doi:10.1007/s12031-013-9956-0.

Wang, Z., Li, Z., Chen, J., Huang, J., Yuan, C., Hong, W., et al. (2012). Association of BDNF gene polymorphism with bipolar disorders in Han Chinese population. *Genes Brain Behav* 11, 524–8. doi:10.1111/j.1601-183X.2012.00797.x.

Wang, Z., Zhang, C., Huang, J., Yuan, C., Hong, W., Chen, J., et al. (2014). MiRNA-206 and BDNF genes interacted in bipolar I disorder. *J Affect Disord* 162, 116–9. doi:10.1016/j.jad.2014.03.047.

Washizuka, S., Ikeda, A., Kato, N., and Kato, T. (2003). Possible relationship between mitochondrial DNA polymorphisms and lithium response in bipolar disorder. *Int. J. Neuropsychopharmacol.* 6, 421–424. doi:10.1017/S1461145703003778.

Yun, D.-H., Pae, C.-U., Drago, A., Mandelli, L., De Ronchi, D., Patkar, A. A., et al. (2008). Effect of the dysbindin gene on antimanic agents in patients with bipolar I disorder. *Psychiatry Investig.* 5, 102–105. doi:10.4306/pi.2008.5.2.102.

Zill, P., Malitas, P. N., Bondy, B., Engel, R., Boufidou, F., Behrens, S., et al. (2003). Analysis of polymorphisms in the alpha-subunit of the olfactory G-protein Golf in lithium-treated bipolar patients. *Psychiatr. Genet.* 13, 65–69. doi:10.1097/01.ypg.0000057881.80011.45.
